# Identification of human–carnivore conflict hotspots to prioritize mitigation efforts

**DOI:** 10.1002/ece3.3565

**Published:** 2017-11-05

**Authors:** Femke Broekhuis, Samuel A. Cushman, Nicholas B. Elliot

**Affiliations:** ^1^ Mara Cheetah Project Kenya Wildlife Trust Nairobi Kenya; ^2^ Wildlife Conservation Research Unit Department of Zoology University of Oxford Recanati‐Kaplan Centre Tubney UK; ^3^ USDA Forest Service Rocky Mountain Research Station Flagstaff AZ USA; ^4^ Mara Lion Project Kenya Wildlife Trust Nairobi Kenya

**Keywords:** conflict hotspots, depredation risk, human–carnivore conflict, husbandry, livestock depredation, Maasai Mara

## Abstract

Human–carnivore conflict is a primary driver of carnivore declines worldwide and resolving these conflicts is a conservation priority. However, resources to mitigate conflicts are limited and should be focused on areas of highest priority. We conducted 820 semistructured interviews with community members living within Kenya's Maasai Mara ecosystem. A multiscale analysis was used to determine the influence of husbandry and environmental factors on livestock depredation inside livestock enclosures (bomas). Areas with a high proportion of closed habitat and protected areas had the highest risk of depredation. Depredation was most likely to occur at weak bomas and at households where there were fewer dogs. We used the results to identify potential conflict hotspots by mapping the probability of livestock depredation across the landscape. 21.4% of the landscape was classified as high risk, and within these areas, 53.4% of the households that were interviewed had weak bomas. *Synthesis and applications*. With limited resources available to mitigate human–carnivore conflicts, it is imperative that areas are identified where livestock is most at risk of depredation. Focusing mitigation measures on high‐risk areas may reduce conflict and lead to a decrease in retaliatory killings of predators.

## 1 INTRODUCTION

Human–carnivore conflict is a primary driver of carnivore declines worldwide (Woodroffe, Thirgood, & Rabinowitz, 2005) and can inflict substantial costs on local communities (Thirgood, Woodroffe, & Rabinowitz, 2005). Large carnivores range widely and their feeding habits pose a direct threat to livestock and people themselves (Packer, Ikanda, Kissui, & Kushnir, 2005). In response to this threat, people commonly kill carnivores (Loveridge, Valeix, Elliot, & Macdonald, 2016), which has resulted in the local extirpation of many carnivore populations (Treves & Karanth, 2003). Throughout the world, human populations are increasing at the edge of protected areas (Wittemyer, Elsen, Bean, Burton, & Brashares, 2008), a dynamic which often results in local carnivore extinctions (Woodroffe, 2000). Resolving and mitigating these conflicts is therefore of primary concern to carnivore conservation and human livelihoods. However, resources to mitigate human–carnivore conflicts are limited and should be focused on areas where conflict risk is highest. It is therefore important to determine which factors influence the likelihood of livestock depredation for mitigation measures to have maximum impact for both carnivores and people.

During the last century, Africa's large carnivores have undergone massive declines, largely due to anthropogenic activity (Ripple et al., 2014). Compared with their historic range, lions (*Panthera leo*) have lost 75% (Riggio et al., 2013), cheetahs (*Acinonyx jubatus*) have lost 91% (Durant et al., 2017), and even highly adaptable and secretive species such as leopards (*P. pardus*) have lost between 63%–75% (Jacobson et al., 2016). Historically, European colonists contributed to these declines (Woodroffe, 2000), while in modern‐day Africa, growing human populations out‐compete carnivores for space and resources (Ripple et al., 2014). By the end of this century, Africa's human population may increase threefold, to between 3.1 and 5.7 billion (Gerland et al., 2014), which will likely cause further carnivore declines (Woodroffe, 2000). Having been eliminated from much of their former range, most African carnivores are now restricted to protected areas and areas of low human density. However, even in these areas, human populations are growing, which will likely result in further range loss, reductions in wild prey and increased human–carnivore conflict (Wittemyer et al., 2008). Furthermore, whether inside or outside protected areas, conflict with humans is often the most common cause of carnivore mortality (Woodroffe & Ginsberg, 1998).

East Africa is home to around 60% of Africa's lions, but populations there are anticipated to decline by as much as 50% over the next two decades, largely due to human–carnivore conflict (Riggio et al., 2013; Woodroffe & Frank, 2005). East Africa's Maasailand is no exception. Maasai pastoralists traditionally keep cattle (*Bos taurus*) and small stock consisting of sheep (*Ovis aries*) and goats (*Capra hircus*). While cattle and small stock both have a monetary value, cattle have a cultural and economic importance (Galaty, 1982) such that depredation of cattle is more likely to result in retaliation against the offending predator (Kissui, 2008). While all large carnivores may be killed in retaliation, lions are more likely to kill cattle compared to other large predators such as spotted hyaenas (*Crocuta crocuta*) or leopards. Coupled with the Maasai cultural traditions of killing lions, this makes lions particularly vulnerable to retaliatory killings in Maasailand (Ikanda & Packer, 2008). For instance, Ikanda and Packer (2008) found that lion killing in the Ngorongoro Crater, Tanzania, is directly proportional to the amount of cattle depredation, and Kissui (2008) found that in the Maasai steppe, 100% of lion attacks resulted in retaliation. While lions are typically the focus of retaliatory killings, leopards, spotted hyaenas, wild dogs (*Lycaon pictus*), and cheetahs are also commonly killed, driving multiple species declines (Inskip & Zimmermann, 2009).

Human–carnivore conflict is determined by both human and carnivore behavior. Human behaviors such as livestock husbandry, which can be deconstructed into herding practices, the structure of livestock enclosures (*bomas*), and the use of deterrents such as dogs (*Canis familiaris*), can determine the likelihood of livestock depredation (Ogada, Woodroffe, Oguge, & Frank, 2003). Concurrently, the general ecology of carnivores, such as social status, habitat‐use, and hunting strategies, may influence their predisposition to livestock depredation (Elliot, Cushman, Macdonald, & Loveridge, 2014; Loveridge et al., 2017). Despite this, human–carnivore conflict is frequently examined from either a human (e.g., Dickman, Hazzah, Carbone, & Durant, 2014) or a carnivore (e.g., Oriol‐Cotterill, Macdonald, Valeix, Ekwanga, & Frank, 2015) perspective. This is because it is often difficult to collect data on predators residing within human‐dominated landscapes, which may alter their behavior to avoid detection (Oriol‐Cotterill et al., 2015). In such cases, environmental variables can be used as a proxy for predator presence and habitat has been used to model livestock loss. For example, Karanth, Gopalaswamy, DeFries, and Ballal (2012) used questionnaire data to spatially map the probability of livestock loss based on environmental variables. The use of such spatial data to identify potential conflict hotspots is becoming increasingly popular in aiding conservation actions (Miller, 2015; Rostro‐García et al., 2016). However, environmental variables are frequently only considered in the immediate vicinity of the depredation event, thereby ignoring the possibility that environmental factors further afield may influence the presence of predators and hence the likelihood of a depredation event to occur (but see Rostro‐García et al., 2016). Recent studies have shown that ecological processes may be driven by environmental factors across a range of spatial scales (Cushman, Elliot, Macdonald, & Loveridge, 2015; Timm, McGarigal, Cushman, & Ganey, 2016) and multiscale approaches should also be considered when determining environmental predictors for human–wildlife conflict (Rostro‐García et al., 2016). Failure to take scale into account may result in an erroneous evaluation of a relationship or detection of a relationship altogether (Cushman & Landguth, 2010) which could misinform management decisions.

We use a multiscale approach to develop a spatial map that models the probability of livestock loss within bomas. More specifically, we conducted 820 semistructured interviews with community members living within and adjacent to wildlife areas in the Maasai Mara ecosystem, Kenya. We had four core objectives: (1) to describe the extent of human–carnivore conflict using self‐reported livestock loss data, (2) to spatially map conflict hotspots by modelling the probability of livestock loss within bomas, (3) to model livestock loss as a function of livestock husbandry, and (4) to identify areas most at risk of livestock depredation based on environmental factors and livestock husbandry.

## 2 METHODS

### 2.1 Study area

**Figure 1:**
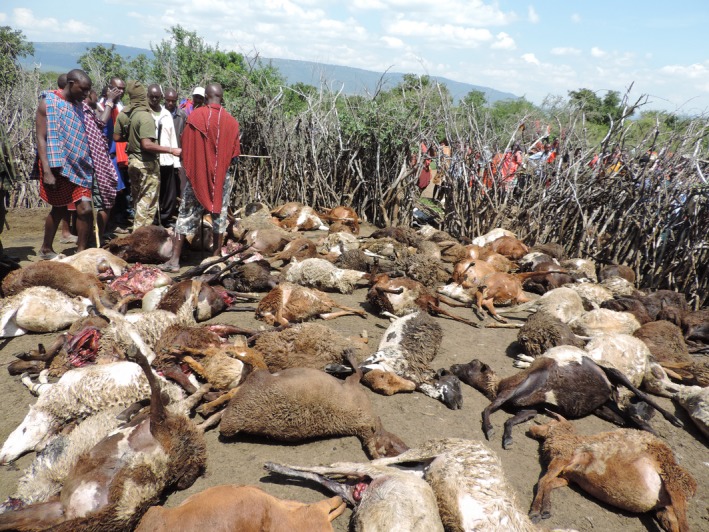
Part of the aftermath of a predator attack on a livestock enclosure (*boma*) in the Maasai Mara, Kenya where more than 200 sheep and goats were killed. Photo credit: Dominic Sakat

The study was conducted in the Maasai Mara landscape in southwestern Kenya (centered at 1°S, 35°E; elevation c. 1,700 m). Data were collected in and around the wildlife areas, which include the Maasai Mara National Reserve and the adjacent wildlife conservancies; Mara Triangle, Mara North, Oloisukut, Ol Chorro, Lemek, Enonkishu, Olare‐Motorogi, Naboisho, Ol Kinyei, and Olarro North (Figure 1). Hereafter, the Maasai Mara National Reserve and the conservancies will collectively be referred to as the protected areas. There are no physical barriers between the protected areas and the surrounding community areas, allowing for free movement of animals. To the south, the Maasai Mara borders the Serengeti National Park in Tanzania, to the north and west it borders intensive agricultural land and east of the Maasai Mara is largely pastoralist settlement (Ogutu, Piepho, Dublin, Bhola, & Reid, 2009). There is a bimodal rainfall pattern with wet seasons between November and December and March to June.

The local community is predominantly Maasai, historically a seminomadic pastoralist society, but now largely sedentary in this area. Their cattle and small stock are generally grazed in the community areas during the day and at night are kept in bomas. Construction of bomas ranges from low (<1 meter), weak barriers made from whistling thorn acacia (*Acacia drepolobium*) branches to tall (>2 meters), strong barriers made from cedar (*Juniperus procera*) posts, placed close together, and surrounded by chain‐link fence (also see Kolowski & Holekamp, 2006).

### 2.2 Data collection

Data were collected through semistructured interviews (Appendix S1) that were conducted in June and July 2015. Ten Maasai men from the community, who had previous experience in conducting questionnaire‐based interviews, were employed to conduct the survey. Before the survey, the interviewers attended a full‐day workshop where they were tested on their interviewing and note‐taking skills and their ability to use a GPS.

As livestock depredation was the focus of the study, interviews were only conducted at households that kept livestock. Prospective interview households were randomly selected using a digitized map of households, created in QGIS 2.8.4 (QGIS Development Team 2015) using high resolution (2.5 m) SPOT 5 imagery (SPOT data/ISIS programme, Copyright CNES). In total, 1,635 households with bomas were mapped and 820 were randomly selected using the *Random selection* tool in QGIS 2.8.4 (QGIS Development Team 2015). Each interviewer was given the coordinates of 82 households. If no household was present at the given coordinates, the interview would be conducted at the closest household with a boma to the initial location. The most senior male of each household was interviewed, as they own the livestock, with the interviewer returning at later date if he was not present. In order to minimize incentives for exaggerating answers, prior to each interview, the interviewee was informed that the survey was independent of the government or any management company and that no compensation would be provided for livestock losses. Consent was given verbally before being interviewed and all respondents agreed to be interviewed.

### 2.3 Extent of human–carnivore conflict

Although a previous study examined human–carnivore conflict at a local level within this system (Kolowski & Holekamp, 2006), no study has quantified the extent of human–carnivore conflict at a broad scale. We therefore asked respondents to provide figures on the number of livestock they own, whether this is more or less compared to 5 years ago and about perceived livestock depredation in the previous 3 months. We chose 3 months as respondents generally bias their answers to recent events (Kissui, 2008). We performed chi‐squared tests to explore variation in depredation events.

### 2.4 Conflict hotspots

To map the probability of livestock depredation, we asked respondents to quantify the perceived number of livestock that had been killed by a predator within their boma during the previous 3 months. We chose only to use losses within bomas as losses within bomas are most likely to result in retaliatory killings (Hazzah, Bath, Dolrenry, Dickman, & Frank, 2017) as these attacks can result in mass deaths and injuries (Figure 1). Additionally, the spatial location of losses outside bomas could not be determined. Although some bomas were attacked several times or several livestock were killed during single attacks, we were primarily interested in whether a depredation event had occurred and therefore this answer was reduced to a binary‐dependent variable. For each household where an interview took place, environmental data were extracted using ArcGIS 10.3 (Environmental Systems Research Institute Inc. 2014). The following independent variables were included in the analyses:

1. Protected area—The protected area network within the study area has a high perimeter:area ratio which may exacerbate edge effects (Woodroffe & Ginsberg, 1998). We therefore hypothesized that the amount of protected area close to a household would be more important in determining livestock depredation than the distance to the closest boundary. The amount of protected area present was calculated using a moving‐window analysis in FRAGSTATS (McGarigal, Cushman, & Ene, 2012) and ranged from 0 (no protected area present) to 1 (completely within a protected area). For each household, this was calculated within the following radii: 90 m, 180 m, 360 m, 720 m, 1,440 m, and 2,880 m.
2. Human presence—We hypothesized that fewer attacks would occur in areas of high human presence as it may be less risky for carnivores to attack livestock further away from human settlement (Loveridge et al., 2016). To quantify human presence, we digitized individual households (see Data collection for details), commercial centers, and towns. To reflect the size of commercial centers and towns, polygons were drawn around each of them which were then converted to points. Using the *point density* function in ArcGIS 10.2.2 (Environmental Systems Research Institute Inc, 2014), the density of human development was calculated at five different scales: 500 m, 1,000 m, 2,000 m, 4,000 m, and 8,000 m.
3. River density—Large carnivores tend to kill more in areas close to water (e.g., Hopcraft, Sinclair, & Packer, 2005), and we hypothesized that this would hold true for livestock depredation. We used the Kernel Density function in ArcGIS 10.3 (Environmental Systems Research Institute Inc, 2014) to calculate river density at six different scales: 90 m, 180 m, 360 m, 720 m, 1,440 m, and 2,880 m.
4. Habitat—We hypothesized that large carnivores would be more likely to predate on livestock in areas with dense vegetation, as they typically select for areas of high catchability (Hopcraft et al., 2005). We created a habitat map (Table S1) and ran a moving‐window analysis in FRAGSTATS (McGarigal et al., 2012) to calculate the proportion of closed habitat within the radii of 90 m, 180 m, 360 m, 720 m, 1,440 m, and 2,880 m for each of the interviewed households. Closed habitat was defined as areas of forest and dense bush that included species such as *Warburgia ugandensis, Acacia xanthophloea*,* Euclea divinorum*,* Croton dichogamus,* and *Tarchonanthus camphoratus* (Table S1).

All the variables, apart from the human presence, were based on data with a spatial resolution of 30 m × 30 m.

The hot spot analysis was conducted in two steps. First, we performed a univariate scaling analysis (e.g., Elliot et al., 2014) for each environmental variable to determine which scale had the strongest relationship with livestock depredation. We used model selection to identify the most supported scale based on Akaike Information Criterion corrected for small sample size (AICc). The scale with the lowest AICc value was inferred to be the one which most strongly influenced the probability of a depredation event occurring in a boma, and thus was retained for the next step. Second, we used generalized linear models (GLMs) with a binomial error structure and a logit function to determine the effect of protected area boundaries, human presence, rivers, and habitat on the probability of livestock being killed inside a boma. The proportion of habitat and protected areas ranged from 0–1, while river density and human presence were both continuous variables. We created a priori candidate models which were ranked using AICc and relative support was assessed using Akaike weights (*wi*). When one model was superior (*w*_*i*_ **> 0.9) this was used, otherwise we averaged parameter estimates across models with AICc differences (Δ_*i*_ **< 2) correcting for model weights (Burnham & Anderson, 2002).

**Figure 2:**
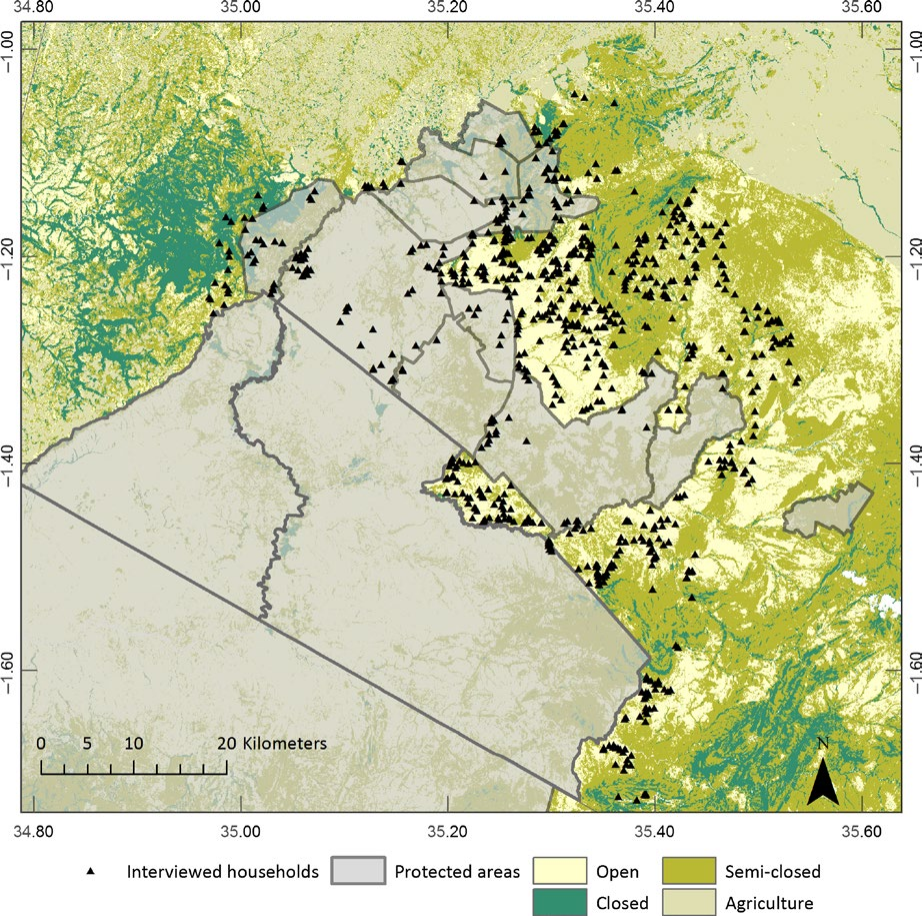
Study extent for the evaluation of livestock depredation. All households within the study area were digitized using satellite imagery and 820 were randomly selected for interviews

Using the results from the second step, we produced a map of conflict hotspots across a wider area of inference, the study extent (Figure 2). Coefficients from the dominant model or those produced by model averaging were used to estimate the relative depredation probability (*p*) for each 30 × 30 m cell across the study area using the following equation in Raster Calculator in ArcGIS 10.3 (Environmental Systems Research Institute Inc, 2014): *p* (x) = exp (z)/(1 + exp (z)).

### 2.5 Livestock husbandry

Respondents were asked how many dogs they kept and whether scarecrows or lion lights (flashing lights believed to deter predators) were used. Having completed the interview, the interviewer inspected the boma. In 19 cases, the bomas were not inspected and these data were excluded from this analysis. To determine the strength of the bomas, variables related to its construction were used which included height (categorical: 0–1 m, 1–2 m, >2 m), construction materials (categorical: branches, wooden posts, cedar posts, predator‐proof boma), number of gates, material used to construct the gates (categorical: branches, wooden frame, wooden poles, metal drum), presence of gaps, and whether there was an outer enclosure present. Each variable was given a code according to its strength where low values indicated weakness and high values indicated strength. For example, for boma height 0–1 m was coded as 1, 1–2 m as 2 and >2 m as 3. Using a nonmetric multidimensional scaling ordination technique, the coded variables were reduced to one independent variable for boma strength.

To model livestock loss as a function of livestock husbandry, we used the same dependent variable as was used to create the conflict hotspots map. We used GLMs with a binomial error structure and a logit function with independent variables consisting of boma strength, number of dogs, and the presence of scare crows and lion lights. We created a priori candidate models and followed the same steps outlined above, using model averaging.

### 2.6 Depredation risk

To identify areas and households most at risk of livestock depredation, and therefore most in need of mitigation measures, we quantified the extent of risky areas and the number of households with poor livestock husbandry within those areas.

## 3 RESULTS

### 3.1 Extent of human–carnivore conflict

**Figure 3:**
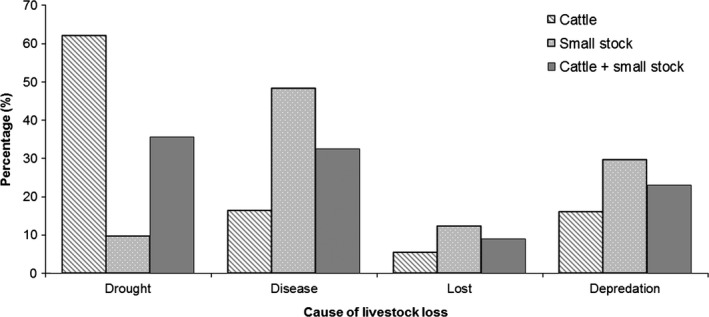
Cause of livestock losses (cattle and small stock) in the Maasai Mara, Kenya

Of the 820 households interviewed, 805 reported to have experienced at least one livestock loss during the previous 3 months, with a total of 37,290 livestock deaths. Of these losses, depredation accounted for 23% (8,551), drought for 36% (13,255), disease for 33% (12,151), and 9% (3,333) of livestock were lost and not found (Figure 3). Most households had lost at least one head of cattle (60%, range 0–100) or at least one head of small stock (67%, range 0–150) to depredation in the previous 3 months. The number of cattle and small stock reportedly kept within the 820 households was 86,599 (range 0–1,600) and 157,018 (range 0–1,200), respectively. With 2,959 cattle and 5,581 small stock lost to depredation, this equates to a loss of 3.4% and 3.6% of respondent's cattle and small stock, respectively, during the previous 3 months. Depredation events were found to occur more frequently when livestock were grazing outside the bomas (74.0%) than when they were kept inside (40.8%; χ_2_ = 3.73, *df* = 1, *p *=* *.05). When asked during which season most livestock are predated on, respondents perceived that the wet season resulted in most depredation of cattle (73%; χ_2_ = 593.2, *df* = 2, *p *<* *.0001) and small stock (59%; χ_2_ = 263.78, *df* = 2, *p *<* *.0001). Compared to 5 years ago, respondents reported to having fewer cows (79%; χ_2_ = 342.24, *df* = 1, *p *<* *.0001). Although most respondents reported to have more (47%) as opposed to fewer (43%) small stock, this was not significant (χ_2_ = 1.138, *df* = 1, *p *=* *.29).

### 3.2 Conflict hotspots

**Figure 4:**
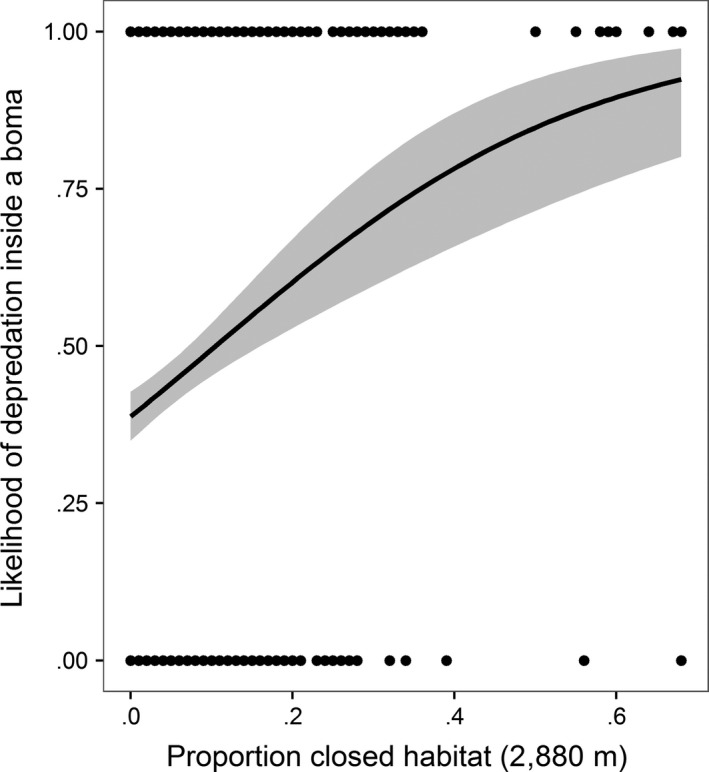
The relationship between the amount of closed habitat present within a 2,880‐m radius of a livestock enclosure (*boma*) and the likelihood that a depredation event would occur inside a boma. Fitted lines are displayed ± 95% confidence intervals

**Figure 5:**
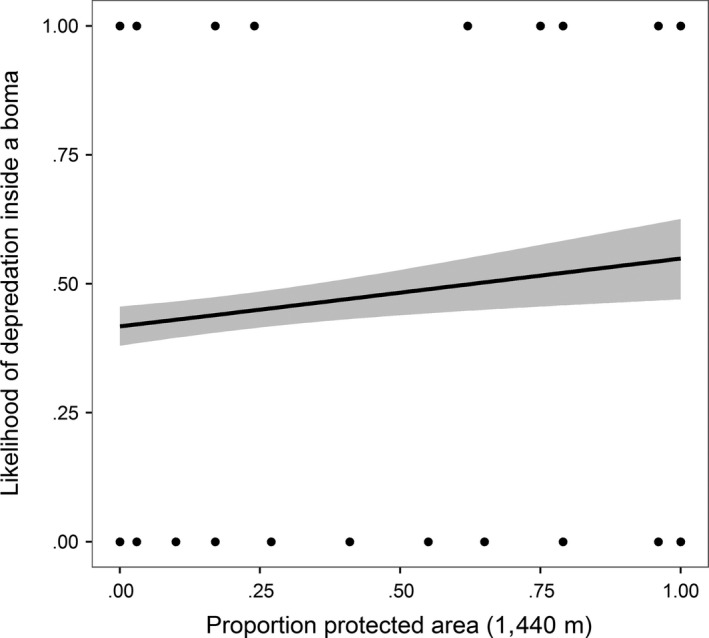
The relationship between the presence of protected areas within a 1,440‐m radius of a livestock enclosure (*boma*) and the likelihood that a depredation event would occur inside a *boma*. Fitted lines are displayed ± 95% confidence intervals

The density of rivers was negatively correlated with the proportion of closed habitat (*t* = −8.0338, *df* = 799, *p *=* *.00) so river density was removed from the analysis. Our univariate scaling analysis showed that environmental variables were most important at broad spatial scales (Table S2). Once the best scale had been identified for each variable, the proportion of closed habitat and the amount of protected area were the best predictors of livestock depredation within bomas (Table S3). Carnivores were most likely to kill livestock in areas where there was a high proportion of closed habitat (estimate = 4.002, CI = 2.266–5.738, Figure 4). In addition, the more protected area that was present the higher the likelihood of a depredation event (estimate = 0.513, CI = 0.111–0.916, Figure 5). The effect of human presence displayed a weak negative relationship with livestock depredation, but this was not significant (estimate = −1.481, CI = −3.364–0.401).

**Figure 6:**
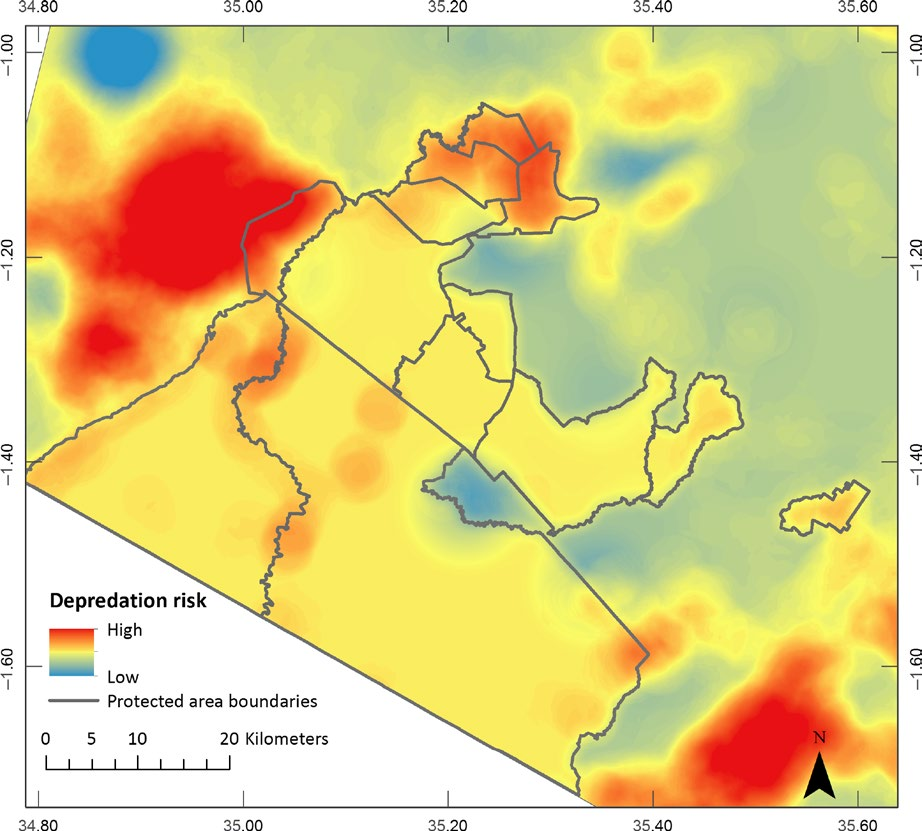
Map depicting the risks of depredation inside a livestock enclosure (*boma*), based on environmental factors, in the Maasai Mara, Kenya

These results were used to spatially map the probability of livestock loss within bomas, which shows highly defined areas of depredation risk both inside and outside the protected areas, most notably the heavily wooded areas to the northwest and southeast of the study area (Figure 6).

### 3.3 Livestock husbandry

**Figure 7:**
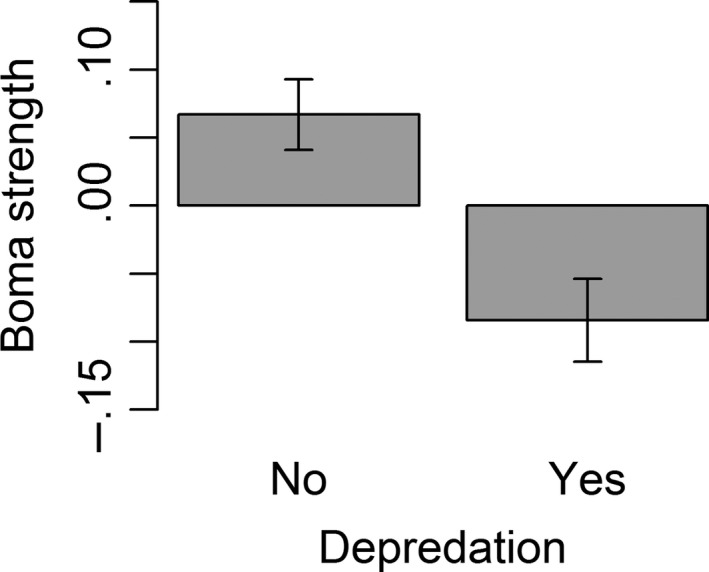
The relationship between boma strength and depredation risk

The top models indicate that, for depredation events that occurred inside a boma, there was a strong negative relationship with boma strength; in other words, the weaker the boma the higher the likelihood of a depredation event (estimate = −0.491, CI = −0.753 to −0.233, Figure 7). Most people kept dogs (n = 760, 94.9%) and the likelihood of a depredation event occurring inside a boma decreased where more dogs were present (estimate = −0.057, CI = −0.110 to −0.007). The presence of other deterrents was less common as scarecrows were seen at 221 (27.6%) bomas and lion lights at 17 (2.1%) bomas. The likelihood of an attack to occur was higher if there were scarecrows (χ_2_ = 11.76, *df* = 1, *p *<* *.00) and lion lights (χ_2_ = 3.83, *df* = 1, *p *=* *.05) present. We tested whether these two deterrents could be confounded by either the presence of dogs or the strength of the boma and we found that scarecrows were more likely to be used at weak bomas (estimate = −0.557, CI = −0.838 to −0.279).

### 3.4 Depredation risk

Households within highly wooded areas, especially those close to, or within, protected areas were most at risk of livestock depredation. In total, 21.40% of the study area was high risk (0.67–1.00), while 78.06% was medium risk (0.34–0.66) and 0.53% was low risk (0.00–0.33; Table 1). While we do not know the locations of all bomas across the study extent, 88 of the interviewed households were situated within high‐risk areas. Of these, 53.41% had weak (strength < 0) bomas and are therefore at extreme risk of livestock depredation (Table 1).

**Table 1:** The level of predation risk within the Maasai Mara, Kenya, and the strength of livestock enclosures (*bomas*) in these areas

Predation risk	Risk category	Area (km^2^)	% Study area	Number of interviewed households	Boma strength	
					Weak (<0)	Strong (>0)
0.00–0.33	Low	38	0.53	0	0	0
0.34–0.66	Medium	5,522	78.06	713	330	383
0.67–1.00	High	1,514	21.40	88	47	41

## 4 DISCUSSION

Our results show that landscape features and husbandry practices are both important predictors of livestock depredation by carnivores inside bomas. The presence of closed habitat, at a broad scale, was positively associated with livestock depredation inside bomas. Predation has been linked with dense vegetation for a variety of carnivores (e.g., Kolowski & Holekamp, 2006; Ogada et al., 2003). With the exception of Rostro‐Garcia et al. (2016), these studies investigated habitat on a fine spatial scale, whereas we show that the presence of closed vegetation has an influence at a much broader scale (2,880 m). For instance, a bomas situated within a small, closed patch of habitat surrounded by open habitat may be at low risk of depredation as compared to a boma situated within an extensive closed habitat, likely due to the amount of concealment afforded to the carnivore. Furthermore, these high‐risk areas of dense vegetation may be ideal habitat for wildlife, and with more land being set aside for wildlife, these areas should be further explored for suitability. Rostro‐Garcia et al. (2016) also found that amount of forest cover predicted depredation risk by leopards and tigers (*P. tigris*) in Bhutan at a broad scale (2,000 m). Similarly, the amount of protected area within the vicinity of a household was an important predictor of livestock depredation. In our study area, the protected area boundaries are extremely irregular, which has resulted in a high perimeter:area ratio, and coupled with the high density of carnivores within the protected areas (Broekhuis & Gopalaswamy, 2016; Elliot & Gopalaswamy, 2017), it is not surprising that our results show that the likelihood of livestock depredation is higher at households with more protected area in the vicinity. It is therefore highly likely that edge effects are occurring within the protected areas, which may be having a negative impact on carnivore populations (Woodroffe & Ginsberg, 1998). This result is also similar to that found by Rostro‐Garcia et al. (2016) in Bhutan who found that risk of livestock depredation by tigers and leopards was high in areas with high edge density between forest and open habitats in proximity to protected areas (area of protected areas within 16 km). This needs further exploration and as new protected areas are established they should look to ensure more regular boundaries and discourage settlement close to protected areas.

Livestock husbandry also influenced the likelihood of livestock loss within a boma. Weaker bomas were more likely to experience depredation by carnivores, a finding which is consistent with numerous studies (e.g., Gusset, Swarner, Mponwane, Keletile, & McNutt, 2009; Ogada et al., 2003). In line with those studies, we recommend improvement of such bomas, especially those that are situated in high‐risk areas within our study area. Furthermore, mitigation efforts could further focus by identifying weak bomas further afield within high‐risk areas across our study extent. The effectiveness of deterrents varied depending on the measure taken. The more dogs present within a household, the lower the likelihood of livestock depredation, consistent with the findings of Ogada et al. (2003). While dogs can be effective deterrents of livestock depredation (Marker, Dickman, & Macdonald, 2005), they also hunt wild prey, compete with wild predators (Wierzbowska, Hędrzak, Popczyk, Okarma, & Crooks, 2016), and can be a reservoir for diseases such as canine distemper virus (CDV) and rabies, which have caused massive die‐offs of a variety of carnivores within this ecosystem (Lembo et al., 2008; Roelke‐Parker et al., 1996). Rabies has been confirmed in 12 carnivore species in the Serengeti National Park in Tanzania (Lembo et al., 2008) and was implicated in the local extinction of wild dogs (Cleaveland et al., 2007), while two CDV epidemics caused large‐scale mortality in lions throughout this ecosystem (Munson et al., 2008). While extensive domestic dog vaccination programmes may have eliminated rabies from some areas, they did not prevent transmission of CDV to carnivores (Viana et al., 2015). Therefore, although domestic dogs may reduce the likelihood of livestock depredation, we caution against promoting them as a mitigation measure in the Maasai Mara.

Unexpectedly, we found that both the presence of lion lights and scarecrows increased the likelihood of livestock depredation, the latter finding being consistent with that of Woodroffe, Frank, Lindsey, ole Ranah, and Romañach (2007). This is likely related to our finding that people with weak bomas used scarecrows, possibly giving a false sense of security. It is also possible that the effectiveness of such deterrents decreases with habituation (Zarco‐González & Monroy‐Vilchis, 2014), or that people experiencing high depredation use such measures, but they are not effective. Further evidence is needed to ascertain their effectivity prior to expending resources on such mitigation measures.

The overall variance explained by the models was low and it is possible that other factors, such as prey abundance, could be important predictors of livestock depredation (Bagchi & Mishra, 2006; Kolowski & Holekamp, 2006), but fine‐scale prey data were not available for the period that the study was conducted. There is a negative relationship between the abundance of wild herbivores and livestock in our study area (Ogutu et al., 2016), and it is possible that in areas where the abundance of wild herbivores is low, predators are more likely to predate on livestock (Khorozyan, Ghoddousi, Soofi, & Waltert, 2015). Another possibility is that because we did not consider predator‐specific differences, the overall result is weaker. Due to the nature of the survey, the interviewers were not able to verify which carnivore species was responsible for an attack, and hence, we grouped all depredation events together. Ideally, predator‐specific risk maps, which account for misidentification (Pillay, Miller, Hines, Joshi, & Madhusudan, 2014), should be produced and combined to create an overall risk map of depredation.

Respondents reported to having lost more livestock to carnivores when grazing their livestock, compared to when they were in a boma. While improving grazing practices may limit financial losses, in terms of carnivore conservation, it may be more efficient to focus on improving bomas, as this is where the retaliatory killing of carnivores is more likely to occur (Hazzah et al., 2017). Overall livestock depredation rates in our study were 3.4% and 3.6% of cattle and small stock, respectively, during a 3 month period. Considering the short time frame, these figures are substantially higher than annual global figures of 0.02–2.6 (Graham, Beckerman, & Thirgood, 2005), and much higher than the 0.6% and 0.2% annual depredation figures for cattle and small stock in a smaller section of our study area (Kolowski & Holekamp, 2006). It is possible that with a rapidly increasing human population (Bedelian, 2014), livestock numbers are also increasing (despite individuals owning fewer cattle), resulting in a dramatic increase in human–carnivore conflict in this area since the study of Kolowski and Holekamp (2006). However, it is equally possible that their smaller study area has lower levels of livestock depredation than across the ecosystem. Whatever the case, livestock depredation rates are high and are likely to result in retaliatory killings, which could drive carnivore declines (Woodroffe et al., 2005). Reinforcing bomas has been proven to reduce depredation and retaliatory killings of carnivores (Hazzah et al., 2014; Lichtenfeld, Trout, & Kisimir, 2014), and our results and conflict hotspots map highlight how limited resources may be put to the most effective use. In summary, we recommend (1) households within and near protected areas and those in dense habitat to have their bomas improved; (2) weak bomas in high‐risk areas be identified and improved as a matter of priority; (3) high‐risk areas may be indictors of good carnivore habitat and, as more areas are being set aside for wildlife with conservancy expansion, these areas should be further explored to ascertain their suitability as wildlife areas; and (4) mitigation measures, such as boma reinforcement or lion lights, be monitored to ensure they are effective at reducing livestock depredation and retaliatory killings.

## CONFLICT OF INTEREST

None declared.

## AUTHOR CONTRIBUTION

FB and NE conceived the study; FB designed methodology and organized data collection; FB, NE, and SC created the GIS layers; FB analyzed the data; FB, NE, and SC interpreted the data; FB and NE led the writing of the manuscript. All authors contributed critically to the drafts and gave final approval for publication.

## References

[ece33565-bib-0001] Bagchi, S. , & Mishra, C. (2006). Living with large carnivores: Predation on livestock by the snow leopard (*Uncia uncia*). Journal of Zoology, 268, 217–224. https://doi.org/10.1111/jzo.2006.268.issue-3

[ece33565-bib-0002] Bedelian, C. E. (2014). Conservation, tourism and pastoral livelihoods: Wildlife conservancies in the Maasai Mara. Kenya: PhD, University College, London.

[ece33565-bib-0003] Broekhuis, F. , & Gopalaswamy, A. M. (2016). Counting cats: Spatially explicit population estimates of cheetah (*Acinonyx jubatus*) using unstructured sampling data. PLoS ONE, 11, e0153875 https://doi.org/10.1371/journal.pone.0153875 27135614 10.1371/journal.pone.0153875 PMC4852905

[ece33565-bib-0004] Burnham, K. P. , & Anderson, D. R. (2002). Model selection and multimodel inference: A practical information‐theoretic approach. New York: Springer‐Verlag.

[ece33565-bib-0005] Cleaveland, S. , Mlengeya, T. , Kaare, M. , Haydon, D. , Lembo, T. , Laurenson, M. K. , & Packer, C. (2007). The conservation relevance of epidemiological research into carnivore viral diseases in the Serengeti. Conservation Biology, 21, 612–622. https://doi.org/10.1111/cbi.2007.21.issue-3 17531040 10.1111/j.1523-1739.2007.00701.x

[ece33565-bib-0006] Cushman, S. , Elliot, N. , Macdonald, D. , & Loveridge, A. (2015). A multi‐scale assessment of population connectivity in African lions (*Panthera leo*) in response to landscape change. Landscape Ecology, 31, 1–17 . https://doi.org/10.1007%2Fs10980-015-0292-3

[ece33565-bib-0007] Cushman, S. , & Landguth, E. (2010). Scale dependent inference in landscape genetics. Landscape Ecology, 25, 967–979. https://doi.org/10.1007/s10980-010-9467-0 10.1111/j.1365-294X.2010.04656.x 20618896

[ece33565-bib-0008] Dickman, A. J. , Hazzah, L. , Carbone, C. , & Durant, S. M. (2014). Carnivores, culture and ‘contagious conflict’: Multiple factors influence perceived problems with carnivores in Tanzania's Ruaha landscape. Biological Conservation, 178, 19–27. https://doi.org/10.1016/j.biocon.2014.07.011

[ece33565-bib-0009] Durant, S. M. , Mitchell, N. , Groom, R. , Pettorelli, N. , Ipavec, A. , Jacobson, A. P. , … Young‐Overton, K. (2017). The global decline of cheetah Acinonyx jubatus and what it means for conservation. Proceedings of the National Academy of Sciences, 114, 528–533. https://doi.org/10.1073/pnas.1611122114 10.1073/pnas.1611122114 PMC5255576 28028225

[ece33565-bib-0010] Elliot, N. B. , Cushman, S. A. , Macdonald, D. W. , & Loveridge, A. J. (2014). The devil is in the dispersers: Predictions of landscape connectivity change with demography. Journal of Applied Ecology, 51, 1169–1178. https://doi.org/10.1111/1365-2664.12282

[ece33565-bib-0011] Elliot, N. B. , & Gopalaswamy, A. M. (2017). Toward accurate and precise estimates of lion density. Conservation Biology, 31, 934–943. https://doi.org/10.1111/cobi.2017.31.issue-4 27958641 10.1111/cobi.12878

[ece33565-bib-0012] Environmental Systems Research Institute Inc (2014). ArcGIS 10.3. Redlands, California, USA.

[ece33565-bib-0013] Galaty, J. G. (1982). Being “Maasai”; being “people‐of‐cattle”: Ethnic shifters in East Africa. American Ethnologist, 9, 1–20. https://doi.org/10.1525/ae.1982.9.1.02a00010

[ece33565-bib-0014] Gerland, P. , Raftery, A. E. , Ševčíková, H. , Li, N. , Gu, D. , Spoorenberg, T. , … Lalic, N. (2014). World population stabilization unlikely this century. Science, 346, 234–237. https://doi.org/10.1126/science.1257469 25301627 10.1126/science.1257469 PMC4230924

[ece33565-bib-0015] Graham, K. , Beckerman, A. P. , & Thirgood, S. (2005). Human–predator–prey conflicts: Ecological correlates, prey losses and patterns of management. Biological Conservation, 122, 159–171. https://doi.org/10.1016/j.biocon.2004.06.006

[ece33565-bib-0016] Gusset, M. , Swarner, M. J. , Mponwane, L. , Keletile, K. , & McNutt, J. W. (2009). Human–wildlife conflict in northern Botswana: Livestock predation by Endangered African wild dog *Lycaon pictus* and other carnivores. Oryx, 43, 67–72. https://doi.org/10.1017/S0030605308990475

[ece33565-bib-0017] Hazzah, L. , Bath, A. , Dolrenry, S. , Dickman, A. , & Frank, L. (2017). From attitudes to actions: Predictors of lion killing by maasai warriors. PLoS ONE, 12, e0170796 https://doi.org/10.1371/journal.pone.0170796 28135338 10.1371/journal.pone.0170796 PMC5279730

[ece33565-bib-0018] Hazzah, L. , Dolrenry, S. , Naughton, L. , Edwards, C. T. T. , Mwebi, O. , Kearney, F. , & Frank, L. (2014). Efficacy of two lion conservation programs in Maasailand, Kenya. Conservation Biology, 28, 851–860. https://doi.org/10.1111/cobi.2014.28.issue-3 24527992 10.1111/cobi.12244

[ece33565-bib-0019] Hopcraft, J. G. C. , Sinclair, A. R. E. , & Packer, C. (2005). Planning for success: Serengeti lions seek prey accessibility rather than abundance. Journal of Animal Ecology, 74, 559–566. https://doi.org/10.1111/j.1365-2656.2005.00955.x

[ece33565-bib-0020] Ikanda, D. , & Packer, C. (2008). Ritual vs. retaliatory killing of African lions in the Ngorongoro Conservation Area. Tanzania. Endangered Species Research, 6, 67–74. https://doi.org/10.3354/esr00120

[ece33565-bib-0021] Inskip, C. , & Zimmermann, A. (2009). Human‐felid conflict: A review of patterns and priorities worldwide. Oryx, 43, 18–34. https://doi.org/10.1017/S003060530899030X

[ece33565-bib-0022] Jacobson, A. P. , Gerngross, P. , Lemeris, J. R. Jr , Schoonover, R. F. , Anco, C. , Breitenmoser‐Würsten, C. , … Dollar, L. (2016). Leopard (*Panthera pardus*) status, distribution, and the research efforts across its range. PeerJ, 4, e1974 https://doi.org/10.7717/peerj.1974 27168983 10.7717/peerj.1974 PMC4861552

[ece33565-bib-0023] Karanth, K. K. , Gopalaswamy, A. M. , DeFries, R. , & Ballal, N. (2012). Assessing patterns of human‐wildlife conflicts and compensation around a central indian protected area. PLoS ONE, 7, e50433 https://doi.org/10.1371/journal.pone.0050433 23227173 10.1371/journal.pone.0050433 PMC3515612

[ece33565-bib-0024] Khorozyan, I. , Ghoddousi, A. , Soofi, M. , & Waltert, M. (2015). Big cats kill more livestock when wild prey reaches a minimum threshold. Biological Conservation, 192, 268–275. https://doi.org/10.1016/j.biocon.2015.09.031

[ece33565-bib-0025] Kissui, B. M. (2008). Livestock predation by lions, leopards, spotted hyenas, and their vulnerability to retaliatory killing in the Maasai steppe, Tanzania. Animal Conservation, 11, 422–432. https://doi.org/10.1111/acv.2008.11.issue-5

[ece33565-bib-0026] Kolowski, J. M. , & Holekamp, K. E. (2006). Spatial, temporal, and physical characteristics of livestock depredations by large carnivores along a Kenyan reserve border. Biological Conservation, 128, 529–541. https://doi.org/10.1016/j.biocon.2005.10.021

[ece33565-bib-0027] Lembo, T. , Hampson, K. , Haydon, D. T. , Craft, M. , Dobson, A. , Dushoff, J. , … Cleaveland, S. (2008). Exploring reservoir dynamics: A case study of rabies in the Serengeti ecosystem. Journal of Applied Ecology, 45, 1246–1257. https://doi.org/10.1111/jpe.2008.45.issue-4 22427710 10.1111/j.1365-2664.2008.01468.x PMC3303133

[ece33565-bib-0028] Lichtenfeld, L. L. , Trout, C. , & Kisimir, E. L. (2014). Evidence‐based conservation: Predator‐proof bomas protect livestock and lions. Biodiversity and Conservation, 24, 1–9 . https://doi.org/10.1007%2Fs10531-014-0828-x

[ece33565-bib-0029] Loveridge, A. J. , Kuiper, T. , Parry, R. H. , Sibanda, L. , Hunt, J. H. , Stapelkamp, B. , … Macdonald, D. W. (2017). Bells, bomas and beefsteak: Complex patterns of human‐predator conflict at the wildlife‐agropastoral interface in Zimbabwe. PeerJ, 5, e2898 https://doi.org/10.7717/peerj.2898 28149682 10.7717/peerj.2898 PMC5267574

[ece33565-bib-0030] Loveridge, A. J. , Valeix, M. , Elliot, N. B. , & Macdonald, D. W. (2016). The landscape of anthropogenic mortality: How African lions respond to spatial variation in risk. Journal of Applied Ecology, 54, 815–825 . https://doi.org/10.1111%2F1365-2664.12794

[ece33565-bib-0031] Marker, L. L. , Dickman, A. J. , & Macdonald, D. W. (2005). Perceived effectiveness of livestock‐guarding dogs placed on Namibian farms. Rangeland Ecology & Management, 58, 329–336. https://doi.org/10.2111/1551-5028(2005)058[0329:PEOLDP]2.0.CO;2

[ece33565-bib-0032] McGarigal, K. , Cushman, S. A. , & Ene, E. (2012). FRAGSTATS v4: Spatial pattern analysis program for categorical and continuous maps. Computer software program produced by the authors at the University of Massachusetts, Amherst. Retrieved from http://www.umass.edu/landeco/research/fragstats/fragstats.html.

[ece33565-bib-0033] Miller, J. B. (2015). Mapping attack hotspots to mitigate human–carnivore conflict: Approaches and applications of spatial predation risk modeling. Biodiversity and Conservation, 24, 1–25 . https://doi.org/10.1007%2Fs10531-015-0993-6

[ece33565-bib-0034] Munson, L. , Terio, K. A. , Kock, R. , Mlengeya, T. , Roelke, M. E. , Dubovi, E. , … Packer, C. (2008). Climate extremes promote fatal co‐infections during canine distemper epidemics in African Lions. PLoS ONE, 3, e2545 https://doi.org/10.1371/journal.pone.0002545 18575601 10.1371/journal.pone.0002545 PMC2435602

[ece33565-bib-0035] Ogada, M. O. , Woodroffe, R. , Oguge, N. O. , & Frank, L. G. (2003). Limiting depredation by african carnivores: The role of livestock husbandry. Conservation Biology, 17, 1521–1530. https://doi.org/10.1111/cbi.2003.17.issue-6

[ece33565-bib-0036] Ogutu, J. O. , Piepho, H. P. , Dublin, H. T. , Bhola, N. , & Reid, R. S. (2009). Dynamics of Mara–Serengeti ungulates in relation to land use changes. Journal of Zoology, 278, 1–14. https://doi.org/10.1111/jzo.2009.278.issue-1

[ece33565-bib-0037] Ogutu, J. O. , Piepho, H.‐P. , Said, M. Y. , Ojwang, G. O. , Njino, L. W. , Kifugo, S. C. , & Wargute, P. W. (2016). Extreme wildlife declines and concurrent increase in livestock numbers in Kenya: What are the causes? PLoS ONE, 11, e0163249 https://doi.org/10.1371/journal.pone.0163249 27676077 10.1371/journal.pone.0163249 PMC5039022

[ece33565-bib-0038] Oriol‐Cotterill, A. , Macdonald, D. , Valeix, M. , Ekwanga, S. , & Frank, L. (2015). Spatiotemporal patterns of lion space use in a human‐dominated landscape. Animal Behaviour, 101, 27–39. https://doi.org/10.1016/j.anbehav.2014.11.020

[ece33565-bib-0039] Packer, C. , Ikanda, D. , Kissui, B. , & Kushnir, H. (2005). Lion attacks on humans in Tanzania. Nature, 436, 927–928. https://doi.org/10.1038/436927a 16107828 10.1038/436927a

[ece33565-bib-0040] Pillay, R. , Miller, D. A. W. , Hines, J. E. , Joshi, A. A. , & Madhusudan, M. D. (2014). Accounting for false positives improves estimates of occupancy from key informant interviews. Diversity and Distributions, 20, 223–235. https://doi.org/10.1111/ddi.2014.20.issue-2

[ece33565-bib-0041] QGIS Development Team (2015). QGIS geographic information system. Open Source Geospatial Foundation Project Retrieved from http://qgis.osgeo.org.

[ece33565-bib-0042] Riggio, J. , Jacobson, A. , Dollar, L. , Bauer, H. , Becker, M. , Dickman, A. , … Pimm, S. (2013). The size of savannah Africa: A lion's (*Panthera leo*) view. Biodiversity and Conservation, 22, 17–35. https://doi.org/10.1007/s10531-012-0381-4

[ece33565-bib-0043] Ripple, W. J. , Estes, J. A. , Beschta, R. L. , Wilmers, C. C. , Ritchie, E. G. , Hebblewhite, M. , … Nelson, M. P. (2014). Status and ecological effects of the world's largest carnivores. Science, 343, 1241484 https://doi.org/10.1126/science.1241484 24408439 10.1126/science.1241484

[ece33565-bib-0044] Roelke‐Parker, M. E. , Munson, L. , Packer, C. , Kock, R. , Cleaveland, S. , Carpenter, M. , … Appel, M. J. G. (1996). A canine distemper virus epidemic in Serengeti lions (*Panthera leo*). Nature, 379, 441–445. https://doi.org/10.1038/379441a0 8559247 10.1038/379441a0 PMC7095363

[ece33565-bib-0045] Rostro‐García, S. , Tharchen, L. , Abade, L. , Astaras, C. , Cushman, S. A. , & Macdonald, D. W. (2016). Scale dependence of felid predation risk: Identifying predictors of livestock kills by tiger and leopard in Bhutan. Landscape Ecology, 31, 1277–1298. https://doi.org/10.1007/s10980-015-0335-9

[ece33565-bib-0046] Thirgood, S. , Woodroffe, R. , & Rabinowitz, A. (2005). The impact of human‐wildlife conflict on human lives and livelihoods (p. 13). People and Wildlife: Conflict or coexistence https://doi.org/10.1017/CBO9780511614774

[ece33565-bib-0047] Timm, B. C. , McGarigal, K. , Cushman, S. A. , & Ganey, J. L. (2016). Multi‐scale Mexican spotted owl (*Strix occidentalis lucida*) nest/roost habitat selection in Arizona and a comparison with single‐scale modeling results. Landscape Ecology, 31, 1209–1225. https://doi.org/10.1007/s10980-016-0371-0

[ece33565-bib-0048] Treves, A. , & Karanth, K. U. (2003). Human‐carnivore conflict and perspectives on carnivore management worldwide. Conservation Biology, 17, 1491–1499. https://doi.org/10.1111/cbi.2003.17.issue-6

[ece33565-bib-0049] Viana, M. , Cleaveland, S. , Matthiopoulos, J. , Halliday, J. , Packer, C. , Craft, M. E. , … Lembo, T. (2015). Dynamics of a morbillivirus at the domestic–wildlife interface: Canine distemper virus in domestic dogs and lions. Proceedings of the National Academy of Sciences, 112, 1464–1469. https://doi.org/10.1073/pnas.1411623112 10.1073/pnas.1411623112 PMC4321234 25605919

[ece33565-bib-0050] Wierzbowska, I. A. , Hędrzak, M. , Popczyk, B. , Okarma, H. , & Crooks, K. R. (2016). Predation of wildlife by free‐ranging domestic dogs in Polish hunting grounds and potential competition with the grey wolf. Biological Conservation, 201, 1–9. https://doi.org/10.1016/j.biocon.2016.06.016

[ece33565-bib-0051] Wittemyer, G. , Elsen, P. , Bean, W. T. , Burton, A. C. O. , & Brashares, J. S. (2008). Accelerated human population growth at protected area edges. Science, 321, 123–126. https://doi.org/10.1126/science.1158900 18599788 10.1126/science.1158900

[ece33565-bib-0052] Woodroffe, R. (2000). Predators and people: Using human densities to interpret declines of large carnivores. Animal Conservation, 3, 165–173. https://doi.org/10.1111/acv.2000.3.issue-2

[ece33565-bib-0053] Woodroffe, R. , & Frank, L. G. (2005). Lethal control of African lions (*Panthera leo*): Local and regional population impacts. Animal Conservation, 8, 91–98. https://doi.org/10.1017/S1367943004001829

[ece33565-bib-0054] Woodroffe, R. , Frank, L. , Lindsey, P. , ole Ranah, S. , & Romañach, S. (2007). Livestock husbandry as a tool for carnivore conservation in Africa's community rangelands: A case–control study. Biodiversity and Conservation, 16, 1245–1260. https://doi.org/10.1007/s10531-006-9124-8

[ece33565-bib-0055] Woodroffe, R. , & Ginsberg, J. R. (1998). Edge effects and the extinction of populations inside protected areas. Science, 280, 2126–2128. https://doi.org/10.1126/science.280.5372.2126 9641920 10.1126/science.280.5372.2126

[ece33565-bib-0056] Woodroffe, R. , Thirgood, S. , & Rabinowitz, A. (2005). The impact of human–wildlife conflict on natural systems. People and Wildlife: Conflict or coexistence. Cambridge University Press https://doi.org/10.1017/CBO9780511614774

[ece33565-bib-0057] Zarco‐González, M. M. , & Monroy‐Vilchis, O. (2014). Effectiveness of low‐cost deterrents in decreasing livestock predation by felids: A case in Central Mexico. Animal Conservation, 17, 371–378. https://doi.org/10.1111/acv.2014.17.issue-4

