# Supplementary material for: Identification of human–carnivore conflict hotspots to prioritize mitigation efforts
Source: Ecol Evol. 2017 Nov 5;7(24):10630–9. doi: 10.1002/ece3.3565 (PMC5743529; doi:10.1002/ece3.3565)
Supplement: Supplementary file 1 [file ECE3-7-10630-s001.pdf]

Information about interview:

Information about the interview and the person conducting the interview

Interviewer name:____

Date:_______________________________________

Start time:_______________________ End time:_____________________________

Household ID:____

Coordinates of household:____

Information about informant:

Information about the person who is being interviewed

Name:

Age: Age set:

Gender: ☐Male ☐Female

Education level: ☐None ☐Primary ☐Secondary ☐Tertiary

Main occupation: ☐Pastoralist ☐Agriculturist ☐Businessman

☐Employed in the tourism sector ☐Other___________________

Religion:☐Christianity ☐Muslim ☐Traditional ☐Other:___________________________

Do you lease land to the conservancy? ☐Yes ☐No

What conservancy are you part of?____

I. Household information

1. How many people in your *olmarei*?
    a. Number of men:________________
    b. Number of women:_______________
    c. Number of children (younger than 18 years):______________

2. Are you the only family in this boma? ☐Yes ☐No
3. If no, how many families use this boma?__________________________

4. In total, how many livestock are in this boma?
    a. Number of cattle:_________________
    b. Number of shoats:________________
    c. Number of donkeys:_______________

II. Livestock

Notes for the person conducting the interview:

- *This section only relates to livestock for which the person being interviewed is responsible, not **all** the livestock in the boma.*

1. How many livestock do **<u>you</u>** have in this boma and why do you keep these livestock?

**Livestock**	**How many do you have?**	**Why do you keep them?**		
		**Sale**	**Subsistence**	**Tradition**
Cattle				
Shoats				
Donkeys				
Chickens				

2. Do you have other livestock somewhere else?☐Yes ☐No

3. Do you now have more or fewer cattle than 5 years ago?

   ☐Fewer ☐ the same ☐more

4. Why do you have fewer/more cattle?____
   ____
   ____

5. Do you now have more or fewer shoats than 5 years ago?

   ☐Fewer ☐ the same ☐more

6. Why do you have fewer/more shoats?____
   ____
   ____

III. Livestock mortalities and losses

Notes for the person conducting the interview:

- *Only ask about mortalities in the last 3 months*

**In the last 3 months how many livestock. . . .**	**Cattle**	**Shoats**	**Donkeys**
Died in total			
Died due to drought			
Died because of disease			
Were lost and not found or stolen			
Depredation			
Person conducting the interview – add the number of deaths and compare to the total given by the person being interviewed to see if number match up			

Notes for the person conducting the interview – if the numbers do not add up, ask why.
Answer:

____
____
____

IV. Husbandry practices

1. Which of the following livestock do you keep **inside** your boma?

☐ Cattle ☐Shoats ☐ Donkeys

2. Do you use any of the following techniques to protect your livestock **while in the boma**:

☐Light fires ☐Scare crow

☐Lion lights ☐Enclosure around the boma

☐Dogs (How many?__________________)

☐Askari (How many?__________________)

☐Other (please specify)___________________________

V. Predators

1. **<u>How many cattle</u>** were killed in the last 3 months?

Predator	During the day		At night	
	In boma	Outside boma	In boma	Outside boma
Lion				
Cheetah				
Leopard				
Spotted hyaena				

2. **<u>How many shoats</u>** were killed in the last 3 months?

Predator	During the day		At night	
	In boma	Outside boma	In boma	Outside boma
Lion				
Cheetah				
Leopard				
Spotted hyaena				

This is the end of the interview. Please thank the participant for their time and collaboration.

End time:_________________________________

VI. Boma construction

After completing the interview please conduct a visual inspection of the boma.

1. What is the height of the boma?

☐0 – 1 metre ☐ higher than 2 meter

☐ 1 – 2 metre ☐Do not know

2. What is the boma made of?

☐Cedar posts ☐Whistling thorn

☐Gum posts ☐Branches

☐Official PPB ☐Other (please specify)____________________________

3. What sort of wire was used for the boma?

☐None ☐ Double twist

☐Single ☐Triple twist

☐ Electric ☐Do not know

4. Number of gates? ________________________

5. What sort of gate does the boma have?

☐Old metal-drum gates ☐Wooden-frame gate

☐Thorn-tree gate ☐Wooden poles gate

6. Can you see any gaps where predators could get in?

☐ Yes ☐ No ☐Unsure

7. Can you see any of the following methods for protection? If so, how many?

☐Scare crow How many?__________________

☐Dogs How many?__________________

☐Lion lights How many?__________________

☐Enclosure around boma

8. Proximity to habitation:

☐0 – 50m ☐ 50-100m

☐100 – 200m ☐More than 200m

Thank you for conducting this interview. Please return this sheet to the Project Community Officers as soon as possible.
