# Supplementary material for: Identification of human–carnivore conflict hotspots to prioritize mitigation efforts
Source: Ecol Evol. 2017 Nov 5;7(24):10630–9. doi: 10.1002/ece3.3565 (PMC5743529; doi:10.1002/ece3.3565)
Supplement: Supplementary file 2 [file ECE3-7-10630-s002.docx]

Table S1: Summary of the different habitat types found in the Maasai Mara, Kenya. Habitat classification was based on three different habitat structures; open, semi-closed and closed*.

| **Habitat structure** | **Habitat type** | **Description** |
| --- | --- | --- |
| **Open** | Bare ground | Areas with no vegetation i.e. rocky outcrops, eroded areas, roads etc. |
|  | Grassland | Open grasslands with the occasional tree or clump of bushes. Grass species incl. Red oat grass (*Themeda triandra*), Thatch grass (*Hyparrhenia rufa*) and Sweet pitted grass (*Bothriochloa insculpa*) and tree species incl. Acacia sp., Balanites |
| **Semi-closed** | Mixed scrub | Acacia and different bushes |
|  | Bushes | Orange-leaved croton (*Croton dichogamus*), Ol Kinyei (*Eulclea divinorum*) |
|  | Whistling thorn | Whistling thorn (*Acacia drepolobium*) |
|  | Acacia woodland | Incl various Acacia sp. Often open understory with tall trees |
| **Closed** | Dense | Dense woodland vegetation. Species incl. *Warburgia ugandensis, Acacia xanthophloea, Euclea divinorum* and *Tarchonanthus camphoratus.* |

* We created a habitat map based on two LandSat 8 images, one from 17^th^ July 2013 and one from 25^th^ January 2014. Both had a 30m spatial resolution. The images were classified based on habitat structure using the Random Forest method, chosen for its high classification accuracy ([Cutler et al. 2007](#_ENREF_8); [Kampichler et al. 2010](#_ENREF_25)). The training data was created in Quantum GIS v2.8.4 ([QGIS Development Team 2015](#_ENREF_51)) using a combination of 378 habitat points obtained on the ground and high resolution SPOT 5 imagery (2.5m resolution) from 2011. In addition to the original satellite images, we also used the Normalised Difference Vegetation Index (NDVI) and texture to increase the accuracy of classification. The classification was carried out using the ‘*randomForest*’ package in the statistical software R 2.14.2 ([R Development Core Team 2016](#_ENREF_52)). Habitat was classified according to three different habitat structures; open, semi-closed and closed. The final map was ground-truthed based on 2000 points created in Quantum GIS v2.8.4 ([QGIS Development Team 2015](#_ENREF_51)) and the final habitat map had an accuracy of 87%.
