# Supplementary material for: Identification of human–carnivore conflict hotspots to prioritize mitigation efforts
Source: Ecol Evol. 2017 Nov 5;7(24):10630–9. doi: 10.1002/ece3.3565 (PMC5743529; doi:10.1002/ece3.3565)
Supplement: Supplementary file 3 [file ECE3-7-10630-s003.docx]

| **Variable** | **Scale (m)** | **LL** | **AICc** | **Δ*i*** | **w*i*** |  |  | **β** | **CI(β)** |
| --- | --- | --- | --- | --- | --- | --- | --- | --- | --- |
|  |  |  |  |  |  |  |  |  |  |
| **Closed habitat** | 2880 | -534.985 | 1074.0 | 0.00 | 0.986 |  |  | 4.340 | 2.688, 6.151 |
|  | 1440 | -539.604 | 1083.2 | 9.24 | 0.010 |  |  | 3.211 | 1.775, 4.785 |
|  | 720 | -540.351 | 1084.7 | 10.73 | 0.000 |  |  | 3.269 | 1.731, 5.002 |
|  | 360 | -544.231 | 1092.5 | 18.49 | 0.000 |  |  | 2.800 | 1.124, 4.748 |
|  | 180 | -546.743 | 1097.5 | 23.52 | 0.000 |  |  | 2.301 | 0.509, 4.500 |
|  | 90 | -548.501 | 1101.0 | 27.03 | 0.000 |  |  | 1.549 | -0.180, 3.599 |
| **Protected areas** | 1440 | -545.033 | 1094.1 | 0.00 | 0.241 |  |  | 0.629 | 0.238, 1.024 |
|  | 720 | -545.051 | 1094.1 | 0.03 | 0.237 |  |  | 0.601 | 0.227, 0.979 |
|  | 360 | -545.154 | 1094.3 | 0.24 | 0.214 |  |  | 0.578 | 0.215, 0.945 |
|  | 180 | -545.529 | 1095.1 | 0.99 | 0.147 |  |  | 0.546 | 0.189, 0.906 |
|  | 90 | -545.730 | 1095.1 | 1.39 | 0.120 |  |  | 0.529 | 0.175, 0.887 |
|  | 2880 | -546.828 | 1097.7 | 3.59 | 0.040 |  |  | 0.540 | 0.121, 0.961 |
| **Human presence** | 8000 | -546.632 | 1097.3 | 0.00 | 0.847 |  |  | -2.397 | -4.254, -0.589 |
|  | 4000 | -549.260 | 1102.5 | 5.26 | 0.061 |  |  | -1.645 | -4.339, 0.941 |
|  | 500 | -549.894 | 1103.8 | 6.52 | 0.032 |  |  | -0.683 | -3.596, 1.843 |
|  | 2000 | -549.958 | 1103.9 | 6.65 | 0.030 |  |  | -0.720 | -4.570, 2.950 |
|  | 1000 | -550.015 | 1104.0 | 6.77 | 0.029 |  |  | -0.297 | -3.804, 3.000 |

**Table S2**. Summary of the univariate scaling analysis. We performed a univariate scaling analysis for each variable to determine the scale that had the strongest relationship with depredation events inside a livestock enclosure in the Maasai Mara, Kenya. We used model selection to identify the most supported scale for each variable based on Akaike Information Criterion corrected for small sample size (AICc). For each variable we retained only the scale with the lowest AICc score for the multivariate analysis. Included are the log likelihood (LL), the AICc values, the AICc differences (Δ*i*) and the Akaike weights (*wi*), in addition to the regression coefficients (β) and their associated 95% confidence interval (CI(β)). A full description of the variables used and their sources can be found in the main text.
