# Supplementary material for: Identification of human–carnivore conflict hotspots to prioritize mitigation efforts
Source: Ecol Evol. 2017 Nov 5;7(24):10630–9. doi: 10.1002/ece3.3565 (PMC5743529; doi:10.1002/ece3.3565)
Supplement: Supplementary file 4 [file ECE3-7-10630-s004.docx]

|  | **Model** | **LL** | **AICc** | **Δ*i*** | **w*i*** |  |
| --- | --- | --- | --- | --- | --- | --- |
|  |  |  |  |  |  |  |
| **Likelihood of a predation event to occur inside a livestock enclosure** | CH_2880_ + Human_8000_ + PA_1440_ | -530.293 | 1068.6 | 0.00 | 0.516 |  |
|  | CH_2880_ + PA_1440_ | -531.613 | 1069.3 | 0.62 | 0.379 |  |
|  | CH_2880_ + Human_8000_ | -533.301 | 1072.6 | 4.00 | 0.070 |  |
|  | CH_2880_ | -534.985 | 1074.0 | 5.35 | 0.036 |  |
|  | Human_8000_ + PA_1440_ | -542.344 | 1090.7 | 22.08 | 0.000 |  |
|  | PA_1440_ | 545.033 | 1094.1 | 25.44 | 0.000 |  |
|  | Human_8000_ | -546.632 | 1097.3 | 28.64 | 0.000 |  |

**Table S3**. Summary of model selection statistics for the Generalized Linear Mixed (GLMs) analysing the likelihood that a predation event would occur inside a livestock enclosure in the Maasai Mara, Kenya. Models were ranked according to the Akaike Information Criterion corrected for small sample size (AICc). Included are the log likelihood (LL), the AICc values, the AICc differences (Δ*i*) and the Akaike weights (*wi*). CH = closed habitat, Human – human presence and PA = protected areas.
